# Feasibility Study of Photoelectrochemical Sensing of Glucose and Urea Using BiVO_4_ and BiVO_4_/BiOCl Photoanodes

**DOI:** 10.3390/s25041260

**Published:** 2025-02-19

**Authors:** Monika Skruodiene, Jelena Kovger-Jarosevic, Irena Savickaja, Jurga Juodkazyte, Milda Petruleviciene

**Affiliations:** Centre for Physical Sciences and Technology, Sauletekio Av. 3, LT-10257 Vilnius, Lithuania; monika.skruodiene@ftmc.lt (M.S.);

**Keywords:** BiVO_4_, BiOCl, photoelectrochemical sensing, glucose, urea, sensitivity, adsorption

## Abstract

**Highlights:**

- Mo-doped BiVO_4_ demonstrated superior glucose and urea sensing with LOD of 0.173 µM and 0.656 µM, respectively.
- The formation of a BiVO_4_/BiOCl heterojunction enhances photoelectrochemical activity; however, it also increases urea and glucose adsorption, raising LOD.

**Abstract:**

This study investigates the photoelectrochemical (PEC) performance of molybdenum-doped bismuth vanadate (Mo-doped BiVO_4_) and its heterojunction with the BiOCl layer in glucose and urea sensing. Photoelectrochemical analyses, including cyclic voltammetry (CV) and electrochemical impedance spectroscopy (EIS), revealed that the formation of a heterojunction enhanced charge carrier separation. The impact of the interaction between the surface of the photoanode and analytes on sensing performance was systematically evaluated. Among the tested configurations, Mo-doped BiVO_4_ exhibited superior glucose sensing with a limit of detection (LOD) of 0.173 µM, while BiVO_4_/BiOCl demonstrated an LOD of 2.474 µM. In the context of urea sensing, Mo-doped BiVO_4_ demonstrated an LOD of 0.656 µM, while BiVO_4_/BiOCl exhibited an LOD of 0.918 µM. Notably, despite the enhanced PEC activity observed in heterostructured samples, Mo-doped BiVO_4_ exhibited superior sensing performance, attributable to good interaction with analytes. The photocurrent response trends—an increase with glucose concentration and a decrease with urea concentration—were attributed to oxidation and adsorption phenomena on the photoanode surface. These findings underscore the critical role of photoanode surface engineering in advancing PEC sensor technology, paving the way for more efficient environmental and biomedical applications.

## 1. Introduction

Environmental pollution poses significant threats to living organisms and ecosystems, necessitating effective monitoring and control of contaminants in wastewater to ensure environmental safety, public health, and regulatory compliance [1,2]. Commonly monitored species in wastewater include ammonium compounds, phosphates, organic pollutants, pharmaceuticals, inorganic ions, salts, and toxic gases [3,4,5]. Advanced detection techniques, such as chromatography (e.g., GC-MS, LC-MS/MS), spectroscopy (e.g., UV-Vis, FTIR, ICP-MS), electrochemical methods (e.g., voltammetry, photoelectrochemical sensors), biosensors, and emerging approaches like Surface-Enhanced Raman Spectroscopy (SERS) and microfluidics, offer high sensitivity and specificity for the identification and quantification of these contaminants, even at trace levels [6,7,8,9].

Photoelectrochemical (PEC) sensors, which uniquely combine light and electrochemical responses to offer high sensitivity, have emerged as promising tools for detecting environmental pollutants [10,11]. Their ability to harness solar energy or external light sources to drive photo-induced reactions enables low-energy operation, distinguishing them from conventional sensors [12]. Compared to traditional techniques like chromatography and fluorescence spectroscopy, PEC sensors provide simpler instrumentation, cost-effectiveness, and the potential for real-time, on-site analysis. The development of PEC sensors is crucial for addressing challenges in monitoring trace-level contaminants, particularly pharmaceuticals, in complex wastewater matrices.

Photoanodes are a critical component in photoelectrochemical (PEC) sensors, as their properties directly affect light absorption, charge separation efficiency, and overall sensor performance. Various semiconductors, including TiO_2_ [13], ZnO [13], WO_3_ [14], and BiVO_4_ [15,16], have been extensively investigated due to their high photoactivity, chemical stability, and environmental compatibility. The choice of photoanode significantly influences the sensitivity, detection limits, and applicability of PEC sensors, as it determines the material’s ability to absorb light and efficiently transport charges.

Despite its promising photoelectrochemical properties, BiVO_4_ suffers from limitations such as low charge carrier mobility, poor conductivity, and rapid recombination of electron-hole pairs, which hinder its efficiency, necessitating further improvements for enhanced performance in practical applications. Doping BiVO_4_ with elements such as tungsten (W), molybdenum (Mo), or other cations has proven to be an effective strategy to enhance its photoelectrochemical performance by improving charge carrier mobility, extending light absorption, and reducing recombination of photogenerated electron-hole pairs [17]. In particular, BiVO_4_-based photoanodes, when combined with other materials such as BiOI, BiOCl, and BiOBr, exhibit enhanced PEC performance [18]. These heterostructures improve charge separation and suppress the recombination of photogenerated carriers.

In this work, heterojunction between BiOCl and Mo-doped BiVO_4_ was developed and investigated for the detection of glucose and urea. Mo doping was selected due to its ability to enhance the photoelectrochemical (PEC) performance of BiVO_4_ by increasing charge carrier density and reducing recombination losses compared to pristine BiVO_4_. Additionally, the formation of a heterojunction with BiOCl was introduced to further improve charge carrier separation, thereby enhancing the overall efficiency of the photoanode. Monitoring glucose and urea levels in wastewater, food, and human samples is essential for assessing both environmental pollution and human health [19,20,21]. By tracking these compounds, we can gain a deeper understanding of the environmental impacts on human health and detect early signs of diseases, thereby enabling more effective public health management and wastewater treatment.

Various types of photoanodes, such as BiOBr/TiO_2_ [22], Ti_3_C_2_/Cu_2_O [23], CdS quantum dots decorated with graphene nanosheets [24], α-Fe_2_O_3_ [25], and CdSe/TiO_2_NTs [26], have already been investigated for glucose detection, as presented in Table 1. However, the number of studies focused on urea detection is limited [27,28]. In this study, the feasibility of glucose and urea sensing using a Mo-doped BiVO_4_/BiOCl heterojunction was analyzed for the first time. Therefore, we believe that the results presented in this work could contribute to the further development and improvement of PEC sensors. Different analytical methods, including X-ray diffraction (XRD), scanning electron microscopy (SEM), cyclic voltammetry (CV), electrochemical impedance spectroscopy (EIS), and chronoamperometry (CA), were employed for the investigation. The photoelectrochemical sensing performance was evaluated using the CA technique in a sodium borate buffer containing varying concentrations of analytes.

## 2. Materials and Methods

### 2.1. Materials

Bismuth (III) nitrate pentahydrate (Bi(NO_3_)_3_ × 5H_2_O) (Carl Roth, Karlsruhe, Germany), ammonium vanadate (NH_4_VO_3_) (Acros Organics, Kandel, Germany), nitric acid (H_3_NO_3_) (Reachmen, Bratislava, Slovakia), ammonium molybdate heptahydrate ((NH_4_)_6_Mo_7_O_24_ × 7H_2_O) (Chempur, Piekary, Slaskie, Poland), citric acid (C_6_H_8_O_6_) (Chempur, Piekary, Slaskie, Poland), polyvinyl alcohol (PVA) (Chempur, Piekary Slaskie, Poland), acetic acid (CH_3_COOH) (Chempur, Piekary Slaskie, Poland), KCl, (Chempur, Piekary Slaskie, Poland), ethylene glycol (Lachner, Neratovice, Czech Republic), boric acid (H_3_BO_3_) (Chempur, Piekary Slaskie, Poland), and sodium hydroxide (NaOH) (Chempur, Piekary Slaskie, Poland) were used as received from suppliers without further purification.

### 2.2. Synthesis of Mo_BiVO_4_ and Mo_BiVO_4_/BiOCl Coatings

Bismuth vanadate (BiVO_4_) coatings were deposited onto a conducting glass substrate (fluorine-doped tin oxide, FTO) using a sol-gel method combined with dip-coating. Initially, 2.94 g of Bi(NO_3_)_3_ × 5H_2_O and 0.702 g of NH_4_VO_3_ (in a 1:1 molar ratio) were dissolved in 23% HNO_3_. Then, 2.52 g of citric acid was added under constant stirring, resulting in a clear blue solution. To achieve the desired sol-gel viscosity, 1 g of PVA and 3 mL of acetic acid were introduced. Finally, 0.7489 g of ammonium molybdate was added to the solution after 4 h of stirring, corresponding to 10 atomic % Mo. Then, the formed sol was stirred for another 24 h at room temperature.

The resulting sol was used to deposit thin films on the FTO substrate. Before coating, the FTO slides (2.5 × 2.5 cm) were thoroughly cleaned in acetone, isopropanol, and water using an ultrasonic bath for 15 min in each solvent. The dip-coating process was performed using a Nadetech ND-DC 11/1 dip-coater (Nadetech Innovations, Noáin, Spain), immersing the FTO at a rate of 100 mm/min into the Mo-doped BiVO_4_ sol. The substrate was kept in the sol for 1 min before being withdrawn at the same rate. The Mo-doped BiVO_4_ coating was annealed in ambient air for 2 h at 450 °C. To increase the coating thickness, the process was repeated twice.

BiOCl layers were formed using a dip-coating process. A solution was prepared by dissolving 2.85 mmol of Bi(NO_3_)_3_·5H_2_O and 3 mmol of KCl in 12.5 mL of ethylene glycol. The transparent solution was stirred magnetically at 50 °C for 2 h. Subsequently, these solutions were used to form the BiOCl layer on Mo-doped BiVO_4_ coatings via the dip-coating technique. The immersion and withdrawal rates were the same as those used during the formation of the Mo-doped BiVO_4_ coatings.

Prepared samples were heated at 160 °C for 24 h in an ambient atmosphere and then allowed to cool to room temperature. To increase the thickness, two layers were formed by repeating the procedure twice. In the manuscript, Mo-doped BiVO_4_ and Mo_BiVO_4_/BiOCl were marked as BiVO_4_ and BiVO_4_/BiOCl, respectively.

### 2.3. Structural and Morphological Analysis

The composition and structure of the synthesized coatings were examined using a SmartLab X-ray diffractometer (Rigaku Corporation, Tokyo, Japan) with a 9 kW rotating copper anode X-ray tube. The analysis was conducted over a 2θ range of 20–80°, utilizing the grazing incidence X-ray diffraction (XRD) method, with a fixed angle (ω) of 0.5° between the parallel X-ray beam and the sample surface. Phase identification was performed using Match software, referencing the Crystallography Open Database (COD). The morphology of the coatings was analyzed using scanning electron microscopy (SEM). Micrographs were taken using an SEM SU-70 (Hitachi Ltd., Tokyo, Japan) with an accelerating voltage of 10 kV and a magnification of 2k. EDX analysis was carried out using a Helios NanoLab dual beam workstation equipped with an EDX spectrometer (Oxford Instruments, Oxfordshire, UK). Mapping and elemental analysis were carried out at 10 kV and 20 kV, respectively.

### 2.4. Photoelectrochemical Investigations

Cyclic voltammetry, electrochemical impedance spectroscopy, open circuit potential (OCP), and chronoamperometry measurements were conducted using a potentiostat/galvanostat Zennium/Zahner Xpot (Zahner Elektrik, Germany) and a three-electrode electrochemical cell. The experiments were conducted in 0.2 M sodium borate buffer (pH~8.5). The buffer solution was prepared by dissolving 12.36 g of boric acid (H_3_BO_3_) in 1 L of distilled water, followed by pH adjustment using 1 M NaOH till the desired pH was reached.

BiVO_4_ and BiVO_4_/BiOCl deposited on FTO substrates were used as working electrodes. A silver/silver chloride electrode with a 3 M KCl solution (Ag/AgCl) and a platinum plate (1 × 0.2 cm^2^) were employed as the reference and counter electrodes, respectively. All potential values stated in this paper are reported versus Ag/AgCl unless indicated otherwise. The potential scan rate of 50 mV s^−1^ was used in CV experiments. The surface of the working electrode (1 cm^2^) was illuminated from the backside (from the FTO side) with an LED Solar Simulator (Redox.me), with a light intensity of 100 mW cm^−2^. The Nyquist plots were measured at 0.5 and 0.7 V for BiVO_4_/BiOCl and BiVO_4_, respectively, with an AC amplitude of 10 mV within a frequency range from 10^4^ to 0.1 Hz under illumination.

### 2.5. Photoelectrochemical Sensing of Glucose and Urea

Photoelectrochemical sensing of glucose and urea was carried out using the chronoamperometry technique in 0.2 M sodium borate buffer containing 1, 5, 15, and 30 mM of glucose or urea. The experiments were conducted in a three-electrode cell, using the same setup as in previous experiments, at an applied potential of 1.2 V. All experiments were repeated 3 times.

### 2.6. Selectivity Experiments

Selectivity experiments were conducted using BiVO_4_ and BiVO_4_/BiOCl photoanodes in a 0.2 M sodium borate buffer solution. Chronoamperometry at 1.2 V was employed to assess the photocurrent response upon sequential additions of glucose and urea. A stock solution of 0.5 M glucose and urea in borate buffer was used, and aliquots of 100 μL or 20 μL were added at 60 s intervals in the following order: 100 μL glucose, 20 μL glucose, 20 μL glucose, 20 μL glucose, 20 μL urea, 20 μL urea, 100 μL urea, 100 μL borate buffer, 100 μL borate buffer, 100 μM glucose, 100 μL glucose, 100 μL urea, 100 μL urea, 100 μL glucose, 100 μL urea, 100 μL sacharose, 100 μL sacharose, 100 μL sacharose, 100 μL lactose, and 100 μL lactose.

## 3. Results and Discussion

### 3.1. Structural and Morphological Analysis of BiVO_4_ and BiVO_4_/BiOCl

**Figure 1:**
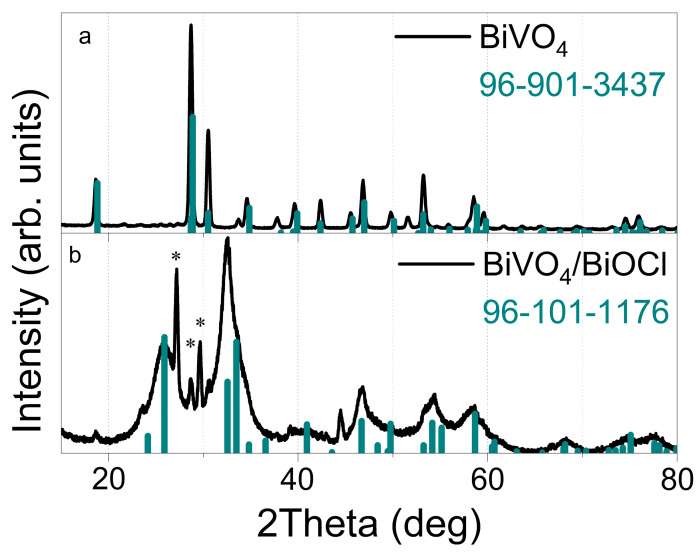
XRD of BiVO_4_ (**a**), BiVO_4_/BiOCl (**b**) coatings. Green columns correspond to indicated references of COD; *—Bi_2_O_3_ (PDF: 96-153-7329).

X-ray diffraction analysis was employed to examine the crystalline structure of the synthesized coatings. In Figure 1a, peaks corresponding to the monoclinic crystalline structure of BiVO_4_ are observed, consistent with the Crystallography Open Database (COD) (PDF: 96-901-3437). EDX elemental mapping images confirming successful Mo-doping of BiVO_4_ are presented in Figure S1. For the BiVO_4_/BiOCl sample, peaks corresponding to the tetragonal crystalline structure of BiOCl are identified, in accordance with PDF: 96-101-1176 (Figure 1b). An additional phase of monoclinic Bi_2_O_3_, consistent with COD (PDF: 96-153-7329), has been identified in the BiVO_4_/BiOCl sample and is indicated by asterisks in the graphs. These XRD results confirm the successful formation of the BiOCl layer on the surface of the BiVO_4_ coating.

**Figure 2:**
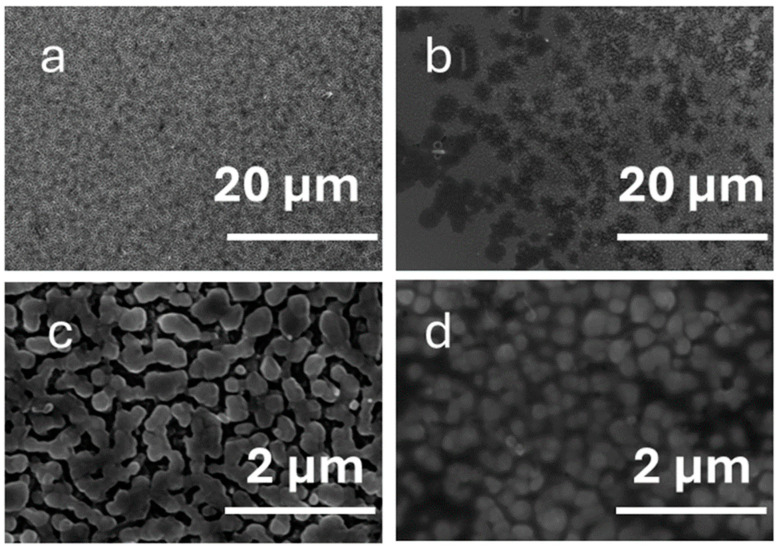
SEM of BiVO_4_ (**a**,**c**) and BiVO_4_/BiOCl (**b**,**d**).

Scanning electron microscopy (SEM) analysis was performed to identify and investigate the morphology of the synthesized coatings (Figure 2). The Mo-doped BiVO_4_ coating consisted of elongated particles interconnected to form a distinctive network-like structure (Figure 2c). The BiVO_4_/BiOCl coating exhibited a morphology similar to BiVO_4_, with a formed film layer covering its surface (Figure 2d). Lower-magnification SEM images clearly illustrated the presence of continuous films developed across the BiVO_4_ surface, indicating successful coating formation and uniformity in both compositions (Figure 2a,b).

### 3.2. Photoelectrochemical Investigations of BiVO_4_ and BiVO_4_/BiOCl Coatings

**Figure 3:**
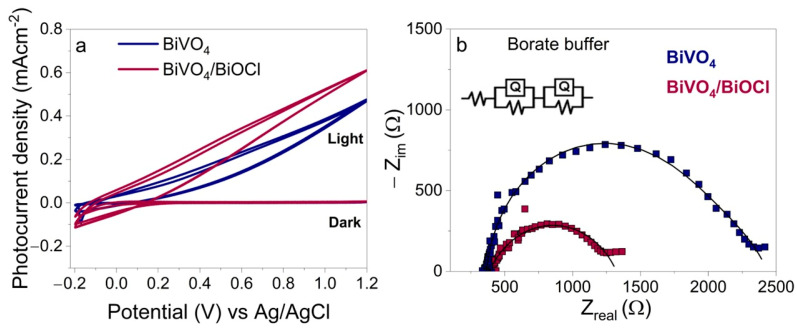
CVs (**a**) and Nyquist plots (**b**) of BiVO_4_ and BiVO_4_/BiOCl photoelectrodes recorded in borate buffer electrolyte under illumination; inset in (**b**) shows equivalent circuit used for fitting the EIS data.

To evaluate the photoelectrochemical activity of the layers, CV measurements were performed in 0.2 M sodium borate buffer (pH~8.5). The results are presented in Figure 3a. The highest photocurrent densities of approximately 0.6 mA cm^−2^ and 0.45 mA cm^−2^ at 1.2 V vs. Ag/AgCl were observed for BiVO_4_/BiOCl and BiVO_4_ coatings, respectively. It can be seen that, in the case of BiVO_4_/BiOCl, photocurrent increased faster, indicating more efficient charge transfer. In order to assess the charge transfer resistance (R*_ct_*) of the samples, the EIS measurements were performed under illumination at 0.7 V in sodium borate buffer (Figure 3b) with BiVO_4_ and BiVO_4_/BiOCl photoanodes. The results demonstrated that the R*_ct_* of BiVO_4_ was ~2250 Ω, while that of BiVO_4_/BiOCl was half as large at ~1250 Ω. These data correlate well with the CV results, indicating improved separation and transportation of charge carriers in the heterostructured photoanode.

### 3.3. Photoelectrochemical Sensing of Glucose and Urea Using BiVO_4_ and BiVO_4_/BiOCl Photoanodes

#### 3.3.1. PEC Sensing of Glucose

**Figure 4:**
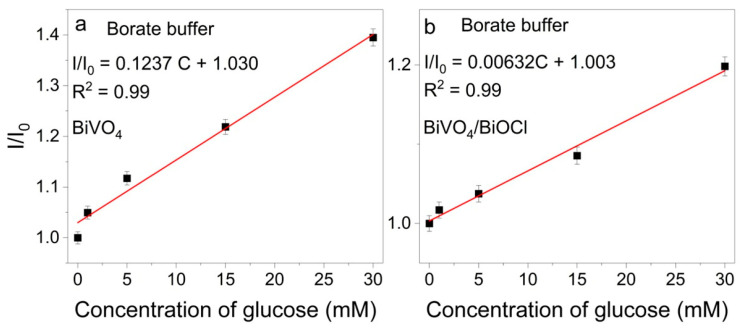
I/I_0_ vs. glucose concentration plots for BiVO_4_ (**a**) and BiVO_4_/BiOCl (**b**) photoelectrodes. Number of replicates: n = 3; the limits of the experimental errors are indicated at the experimental points.

The photoelectrochemical sensing of glucose was investigated with BiVO_4_ and BiVO_4_/BiOCl photoanodes in sodium borate buffer (pH~8.5) in the absence or presence of 1, 5, 15, or 30 mM of glucose, using the chronoamperometry technique. The resulting chronoamperograms are displayed in Figure S2. The current density ratios (I/I_0_, where I and I_0_ stand for photocurrent in solution with and without glucose, respectively) were calculated by measuring the photocurrent density at the midpoint of the second step of chronoamperograms presented in Figure S2. The investigation revealed that I/I_0_ increased with rising glucose concentrations (Figure 4a,b), presumably due to oxidation of glucose by the photogenerated holes formed on the surface of the photoanode [30,31]. The standard glucose oxidation potential, E^0^, is defined as 0.05 V (SHE) [32,33], whereas the energy of photogenerated holes in BiVO_4_ is approximately 2.5 V [34]. Therefore, holes have sufficient energy to drive direct glucose oxidation Additionally, indirect glucose oxidation can occur through the reaction with radical species (e.g., HO^∙^), formed in the interaction of holes with water molecules.

In Figure 4a,b, the linear tendency of I/I_0_ vs. glucose concentration is observed, thus indicating the suitability of these coatings for evaluation of the limit of detection (LOD) and sensing abilities. Given the observed alterations in the photocurrent density and the varying attainment of stabilization across different samples, as shown in Figure S2, the I/I_0_ vs. glucose concentration regression equations and limit of detection (LOD) were evaluated at 5-s intervals during the second pulse of CA, commencing from the 10th second post-illumination. The results of this analysis are presented in Figure S3 in the graphs of I/I_0_ vs. glucose concentration. The regression equations, correlation coefficients, and the calculated limit of detection (LOD) are documented in Table S1, categorized by CA time. The limit of detection (LOD) was calculated based on 3σ/S, in which σ is the standard deviation of a blank signal, and S is the slope of the linear calibration plot presented in Figure S3. The data demonstrate that the LOD for the BiVO_4_ coating ranges from 0.173 to 1.804 µM, and for the BiVO_4_/BiOCl coating, the LOD ranges from 2.474 to 3.103 µM. As illustrated in Table S1, the LOD value depends on the position at which the photocurrent measurements are taken. The lowest limit of detection (LOD) was calculated to be 0.173 µM and 2.474 µM for BiVO_4_ and BiVO_4_/BiOCl, respectively, with the regression equations I/I_0_ = 0.1237 C + 1.030 and I/I_0_ = 0.00632 C + 1.003 for BiVO_4_ and BiVO_4_/BiOCl, respectively.

A comparison of the presented results with those from the existing literature shows that the detection limit of glucose using BiVO_4_ photoanode falls within the range of reported values, i.e., 0.73 μM [35], 0.13 μM [15], and 0.12 μM [36]. It is evident that the results obtained in this study align with the aforementioned findings, suggesting consistency in the performance of the photoanode. The formation of a heterojunction has been demonstrated to enhance photoelectrochemical activity and facilitate the separation of charge carriers; however, it has not improved the sensing properties.

#### 3.3.2. PEC Sensing of Urea

**Figure 5:**
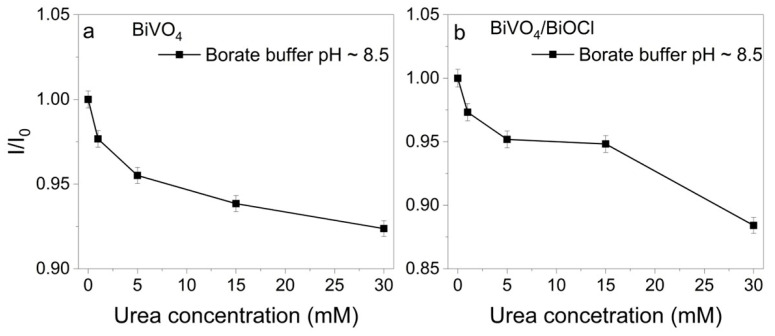
I/I_0_ vs. urea concentration plots for BiVO_4_ (**a**) and BiVO_4_/BiOCl (**b**) photoelectrodes in sodium borate buffer. Number of replicates: n = 3.

**Figure 6:**
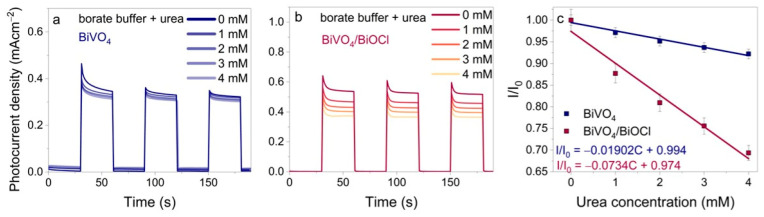
Chronoamperograms of BiVO_4_ (**a**) and BiVO_4_/BiOCl (**b**) photoelectrodes recorded in borate buffer without or with urea at 1.2 V; linear relationship between I/I_0_ and urea concentration for BiVO_4_ and BiVO_4_/BiOCl (**c**).

Analogous experiments were performed in 0.2 M sodium borate buffer containing 1–30 mM of urea. The recorded chronoamperograms are presented in Figure S4. The plots of I/I_0_ vs. urea concentration are shown in Figure 5, where contrasting results are evident compared to those observed with glucose. In alkaline electrolytes (pH 14), the standard urea oxidation potential is E^0^ = −0.46 V vs. SHE (E^0^ = −0.14 V at pH = 8.5). Therefore, photogenerated holes have sufficient energy to drive the urea oxidation reaction, like in the case of glucose [37,38,39]. In addition, indirect oxidation reactions of urea are possible, in which urea reacts with formed radicals or intermediates [37,40]. Similar principles are used in other photoelectrochemical sensors with different types of analytes [41,42]. In general, when urea reacts with holes, an increase in photocurrent should be expected, like in the photoanodic oxidation of glucose. However, the photocurrent in the borate buffer decreased (Figure 5). The examination of the curves obtained in sodium borate buffer reveals a linear correlation for all samples at lower concentrations of urea, with a saturation observed at a concentration of 5 mM. Consequently, additional experiments were conducted with lower concentrations of urea, ranging from 1 to 4 mM, to further investigate this phenomenon (Figure 6). A decrease in photocurrent was observed, indicating a potential relationship between urea concentration and the photocurrent response. The linear correlation between I/I_0_ and the concentration of urea is evident in Figure 6c. The regression equations for BiVO_4_ and BiVO_4_/BiOCl are obtained as follows: I/I_0_ = −0.01902 C + 0.994 (R^2^ = 0.98) and I/I_0_ = −0.0734 C + 0.974 (R^2^ = 0.97), respectively. The calculated LODs for the BiVO_4_ and BiVO_4_/BiOCl coatings were determined to be 0.656, 0.918, and 1.69 μM, respectively. It is noteworthy that the BiVO_4_ coating exhibited a lower LOD, a characteristic that was also observed in the glucose case.

#### 3.3.3. Evaluation of Glucose and Urea Adsorption on the Surface of Photoanodes

**Figure 7:**
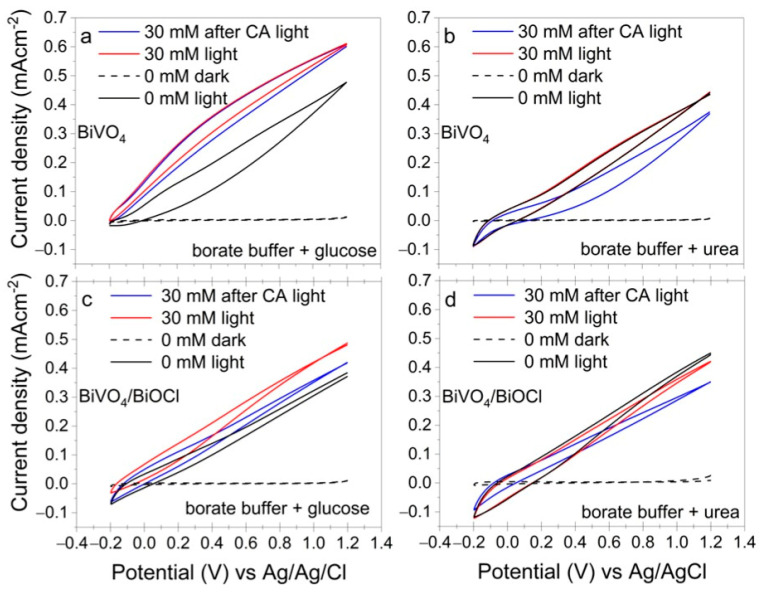
CVs of BiVO4 (**a**,**b**) and BiVO_4_/BiOCl (**c**,**d**) photoelectrodes in sodium borate buffer containing glucose (**a**,**c**) and urea (**b**,**d**).

In general, the photoanodic oxidation of organic compounds is expected to increase the photocurrent. In order to find out the possible reasons for the photocurrent decrease in urea-containing electrolytes, the adsorption of analytes on the surface of the photoelectrodes was studied. Cyclic voltammograms (CVs) were recorded for all coatings in blank electrolytes (sodium borate buffer) and in electrolytes containing 30 mM of either glucose or urea. Then, BiVO_4_ and BiVO_4_/BiOCl samples were polarized at 1.2 V under illumination for 10 min (CA experiment), and subsequently CVs were recorded to evaluate possible passivation of the surface. The results are presented in Figure 7.

As can be seen in Figure 7a, the photocurrent of BiVO_4_ increased in the electrolyte containing glucose as compared to the blank solution, with no significant changes observed after 10 min of the CA experiment. The BiVO_4_/BiOCl coating also exhibited an increase in photocurrent in the presence of glucose but showed a decrease in photocurrent after CA, indicating some changes in the photoanode surface state (Figure 7b). This could be related to the adsorption of glucose oxidation products, decreasing the electrochemically active surface area of the photoelectrode. This behavior likely explains the lower limit of detection (LOD) observed for the BiVO_4_/BiOCl coating compared to the BiVO_4_ coating. A comparison of BiVO_4_ and BiVO_4_/BiOCl coatings reveals that the photocurrent exhibits a more pronounced increase for the BiVO_4_ in a buffer with glucose, suggesting a more favorable glucose oxidation reaction.

The CVs of BiVO_4_ and BiVO_4_/BiOCl samples in electrolytes containing 30 mM of urea appear very similar to those recorded in pure sodium borate buffer solution. However, after the CA experiment, a decrease in photocurrent was observed (Figure 7b,d), suggesting surface fouling caused by urea or its oxidation products, which hinder charge transfer and passivate the surface.

**Figure 8:**
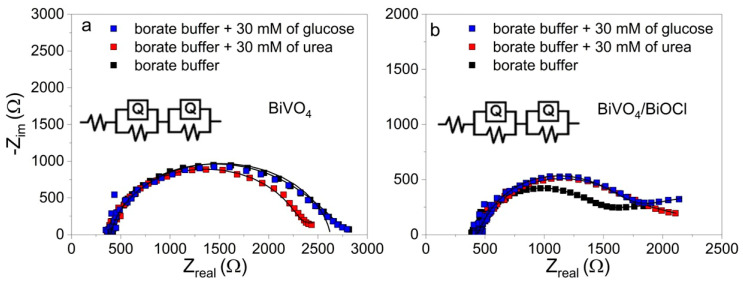
Nyquist plots of BiVO_4_ (**a**) and BiVO_4_/BiOCl (**b**) photoelectrodes recorded in sodium borate buffer in the absence or presence of 30 mM of glucose or urea.

To confirm the influence of the adsorption of glucose and urea or their oxidation products on the surface of the photoanode, EIS measurements were performed with BiVO_4_ and BiVO_4_/BiOCl photoelectrodes under illumination at 0.5 V in electrolytes without and with 30 mM of either glucose or urea (Figure 8). Higher concentrations of the analytes were used in order to make the concentration effect on the R*_ct_* of BiVO_4_ and BiVO_4_/BiOCl coatings more pronounced. As can be seen from the Nyquist plots in Figure 8, in the case of BiVO_4_, the R*_ct_* in the glucose-containing electrolyte was marginally lower in comparison to the blank borate buffer or the electrolyte with urea. This result confirms the idea that the PEC oxidation of glucose on the surface of BiVO_4_ is more efficient. However, for the BiVO_4_/BiOCl sample, the presence of both glucose and urea in the electrolyte resulted in an increase in R*_ct_*, implying possible adsorption of urea, glucose, or their oxidation products, which reduces the electrochemically active surface area and inhibits oxidation reactions at the photoelectrode/electrolyte interface.

Furthermore, the effect of glucose and urea on the PEC performance of the photoelectrodes was studied using open-circuit potential (OCP) measurements in the dark and under illumination (Figure S5). In the case of BiVO_4_ photoelectrode, the addition of either glucose or urea led to an increase in OCP values in the dark, which was particularly evident during the first minute of measurement (Figure S5a,b), whereas the response of the OCP of BiVO_4_/BiOCl to both analytes was the opposite—the potential decreased, especially in the presence of urea (Figure S5c,d). This observation indicates the different nature of the interaction between photoelectrode and analyte.

**Figure 9:**
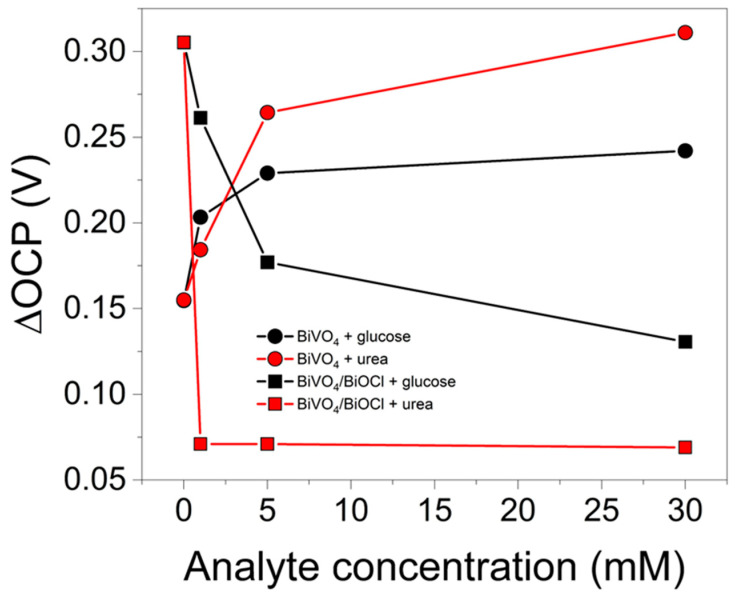
ΔOCP vs. concentration of analyte for BiVO_4_ and BiVO_4_/BiOCl photoelectrodes measured in sodium borate buffer in the absence or presence of glucose or urea.

Under illumination, the OCP of both photoelectrodes shifted towards more negative values, which is a typical behavior of n-type semiconductors. However, the magnitude of this shift (ΔOCP) was dependent on the concentration of the analyte and differed significantly for BiVO_4_ and BiVO_4_/BiOCl photoelectrodes. ΔOCP was calculated as the average of the difference between the OCP values in darkness and under illumination read at the end of the dark/light periods of the third, fourth, and fifth steps in the chronopotentiogram (Figure S5). The results of ΔOCP variation are summarized in Figure 9. While ΔOCP of BiVO_4_ increased with an increase in glucose and urea concentration, the trend for BiVO_4_/BiOCl heterojunction was the opposite, highlighting distinct photoelectrochemical behaviors in the tested electrolytes. It is noteworthy that in the case of BiVO_4_/BiOCl in the urea-containing solution, ΔOCP was the smallest and almost independent of the urea concentration. In general, a smaller shift of OCP would imply a faster hole transfer, which prevents the accumulation of charges and stabilizes the surface potential of the photoelectrode. With faster hole transport, an increase in photocurrents with an increase in analyte concentration should be expected. However, the opposite trend is observed (Figure 6). Such a phenomenon can only be explained by the excessive adsorption of urea or glucose, or the products of their PEC decomposition, which block active surface sites, increase recombination, and passivate the photoanode surface, reducing its PEC activity.

These results highlight the critical role of photoanode surface–analyte interactions in determining the performance of a PEC system. Though the band alignment within BiVO_4_/BiOCl heterojunction facilitates the separation of photogenerated charge carriers, as discussed in detail in our previous study [43], data reported herein demonstrates that there is a complex interplay between charge transfer dynamics, surface passivation, and recombination processes.

#### 3.3.4. Selectivity Tests of BiVO_4_ and BiVO_4_/BiOCl

**Figure 10:**
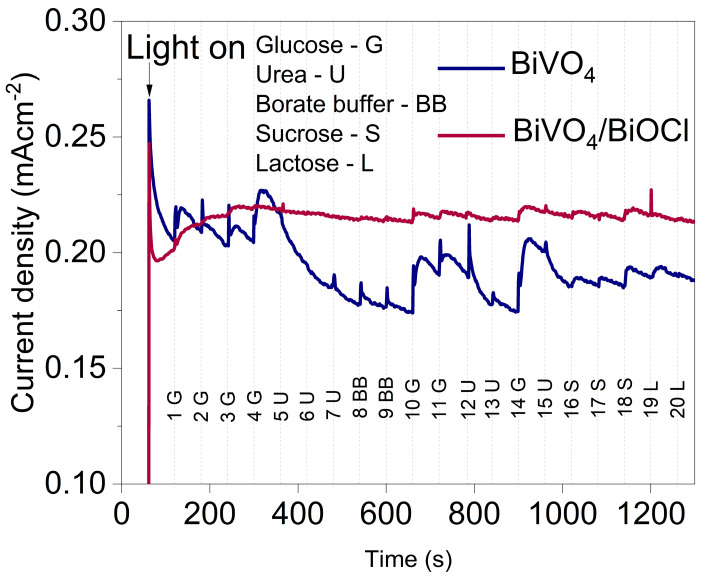
Selectivity performance of BiVO_4_ and BiVO_4_/BiOCl photoanodes in 0.2 M sodium borate buffer, evaluated using sequential additions of indicated analytes during chronoamperometric measurements at 1.2 V.

Selectivity experiments were conducted using BiVO_4_ and BiVO_4_/BiOCl photoanodes, and the results are presented in Figure 10. Sequential additions of glucose urea, borate buffer, saccharose, and lactose were made at 60 s intervals near the surface of the photoanode in the following order: 100 μL glucose (step 1), 20 μL glucose (step 2), 20 μL glucose (step 3), 20 μL glucose (step 4), 20 μL urea (step 5), 20 μL urea (step 6), 100 μL urea (step 7), 100 μL borate buffer (step 8), 100 μL borate buffer (step 9), 100 μL glucose (step 10), 100 μL glucose (step 11), 100 μL urea (step 12), 100 μL urea (step 13), 100 μL glucose (step 14), 100 μL urea (step 15), 100 μL sacharose (steps 16-18) and 100 μL lactose (steps 19–20). For the BiVO_4_ photoanode, a strong response to glucose was observed. After the addition of 100 μL of glucose (Step 1), the photocurrent increased by approximately 7%, from 0.205 mA cm^−2^ to 0.22 mA cm^−2^. Upon the addition of 20 μL of glucose (Step 2), the photocurrent increased proportionally to the concentration, following the trend established in Step 1. Steps 5–7 in the graph correspond to the addition of 20 μL portions of the urea-containing solution, and a gradual decrease in the photocurrent was observed. Steps 8–9 represent the addition of a blank borate buffer, confirming the stable operation of the photoanode. In steps 14–15, a significant increase in photocurrent was observed upon glucose addition, followed by a sharp decrease upon urea addition. Steps 16–17 correspond to addition of sucrose, where a small increase in photocurrent can be observed, as well as in the case of lactose (Steps 19–20). The obtained results demonstrate the high selectivity of BiVO_4_ toward glucose and urea. For the BiVO_4_/BiOCl photoanode, a similar trend was observed; however, the photocurrent changes were significantly lower compared to BiVO_4_ coating. Weaker photocurrent variations are consistent with higher LODs for glucose and urea obtained with BiVO_4_/BiOCl photoanode. 

Semiconductor-based PEC sensors rely on light-induced separation of charge carriers and subsequent electrochemical reactions to generate a measurable electrical signal, i.e., photocurrent. In a pure electrolyte under conditions of light exposure, holes photogenerated in BiVO_4_ or BiVO_4_/BiOCl photoanodes oxidize water or electrolyte ions. In the presence of glucose or urea, these organic compounds compete with water molecules for the interaction with holes. The oxidation potential of organic compounds is usually lower than that of water molecules, making this process thermodynamically more favorable. Therefore, an increase in photocurrent upon the addition of organic analytes is expected, and was demonstrated to occur in glucose-containing electrolytes in PEC systems with BiVO_4_ and BiVO_4_/BiOCl. The adsorption of reacting species on the photoelectrode surface plays a very important role, but it can either facilitate or hinder the PEC processes, as was found in the photooxidation of urea. Strong adsorption of urea or the products of its oxidation, most likely, passivates and blocks the surface, resulting in kinetic limitation of PEC reactions and a decrease in photocurrent with an increase in urea concentration. The chemical nature of the photoelectrode and the analyte determines the strength of the adsorptive interaction.

## 4. Conclusions

In this study, the applicability of Mo-doped BiVO_4_ and heterostructured BiVO_4_/BiOCl photoelectrodes for photoelectrochemical sensing of glucose and urea was investigated. Cyclic voltammetry and electrochemical impedance spectroscopy measurements indicated that the formation of a heterojunction enhanced the separation of charge carriers. However, sensing performance was found to be strongly influenced by the nature of the semiconductor, as well as the nature of the analyte itself. The best performance for glucose sensing was demonstrated by BiVO_4_ with an LOD = 0.173 µM, whereas for BiVO_4_/BiOCl photoanode, the LOD was 2.474 µM. In the case of urea, LODs were found to be 0.656 and 0.918 µM for the BiVO_4_ and BiVO_4_/BiOCl coatings, respectively. The experimental findings demonstrated that both photoelectrodes exhibited a decrease in photocurrent with an increase in urea concentration. On the basis of CV, EIS, and OCP measurements, this phenomenon was attributed to the adsorption of urea or its oxidation products on the surface of the photoanode. This led to partial blocking of the electrochemically active surface and inhibition of photoanodic oxidation reactions at the photoelectrode/electrolyte interface. Satisfactory selectivity of Mo-doped BiVO_4_ for glucose and urea was demonstrated. It is anticipated that these findings will contribute to the advancement of PEC sensor development.

## Figures and Tables

**Table 1:** Literature overview of photoanodes for PEC glucose sensors.

Photoanode	Limit of Detection (LOD)	Reference
BiOBr-TNTA	0.010 μM	[22]
CdS quantum dots	7 μM	[24]
Ti_3_C_2_/Cu_2_O	0.00017 μM	[23]
α-Fe_2_O_3_	0.05 μM	[25]
CdSe/TiO_2_NTs Heterojunction	3.1 μM	[26]
BiVO4	0.13 μM	[15]
BiVO_4_ with gold nanoparticles	0.00026 μM	[29]

## Data Availability

The data presented in this study are available on request from the corresponding author.

## Supplementary Materials

The following supporting information can be downloaded at: https://www.mdpi.com/article/10.3390/s25041260/s1, Figure S1: EDX mapping of Mo-doped BiVO4; Figure S2: Chronoamperograms of BiVO_4_ (a) and BiVO_4_/BiOCl (b) coatings recorded in 0.2 M borate buffer without and with 1, 5, 15, and 30 mM of glucose in the dark and under light illumination with 30s intervals. Applied potential 1.2 V vs. Ag/AgCl; Figure S3: I/I_0_ vs. glucose concentration of BiVO4 (a) and BiVO4/BiOCl (b) samples; Figure S4: Chronoamperograms of BiVO_4_ (a) and BiVO_4_/BiOCl (b) coatings recorded in 0.2 M borate buffer without and with 1, 5, 15, and 30 mM of urea in the dark and under light illumination with 30 s intervals. Applied potential 1.2 V vs. Ag/AgCl; Figure S5: Variation of the open circuit potential (OCP) of BiVO4 (a,b) and BiVO4/BiOCl (c,d) coatings in 0.2 M borate buffer without and with 1, 5 and 30 mM of glucose (a,c) or urea (b,d); Table S1: Linear regression equations, correlation coefficients, and LOD of BiVO_4_ and BiVO_4_/BiOCl samples.

## Abbreviations

The following abbreviations are used in this manuscript:

CV	Cyclic voltammetry, cyclic voltammogram
EIS	Electrochemical impedance spectroscopy
LOD	Limit of detection
PEC	Photoelectrochemical
XRD	X-ray diffraction
SEM	Scanning electron microscopy
CA	Chronoamperometry
COD	Crystallography open database
SHE	Standard hydrogen electrode
