# Supplementary material for: Feasibility Study of Photoelectrochemical Sensing of Glucose and Urea Using BiVO4 and BiVO4/BiOCl Photoanodes
Source: Sensors (Basel). 2025 Feb 19;25(4):1260. doi: 10.3390/s25041260 (PMC11861178; doi:10.3390/s25041260)
Supplement: Supplementary file 1 [file sensors-25-01260-s001.zip › sensors-3481382-supplementary.pdf]

Feasibility Study of Photoelectrochemical Sensing of Glucose and Urea Using $BiVO_4$ and $BiVO_4$/BiOCl Photoanodes

Monika Skruodiene, Jelena Kovger-Jarosevic, Irena Savickaja, Jurga Juodkazyte and Milda Petruleviciene *

Centre for Physical Sciences and Technology, Sauletekio Av. 3, LT-10257 Vilnius, Lithuania; monika.skruodiene@ftmc.lt (M.S.); jelena.kovger@ftmc.lt (J.K.-J.); irena.savickaja@ftmc.lt (I.S.); jurga.juodkazyte@ftmc.lt (J.J.)

* Correspondence: milda.petruleviciene@ftmc.lt

Supporting information

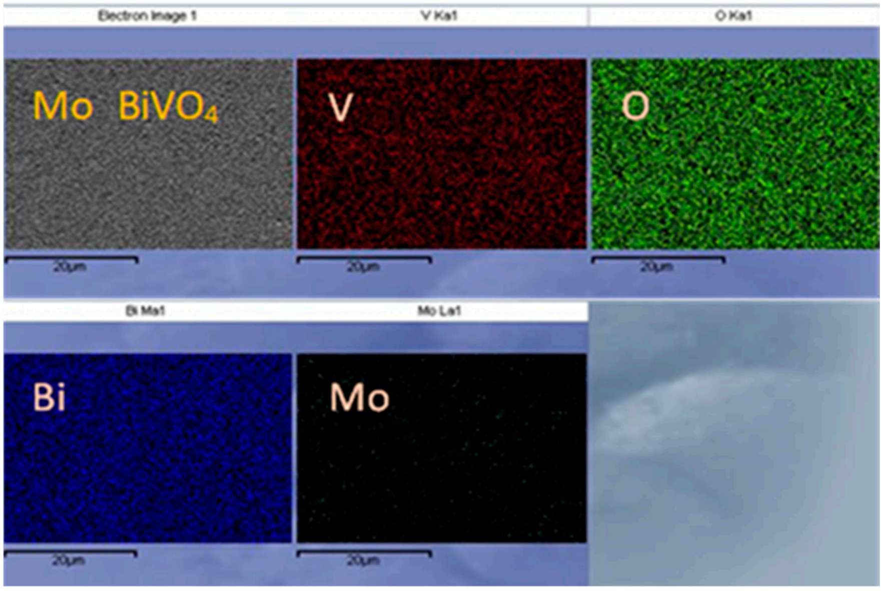

Figure S1. EDX elemental mapping image of Mo-doped $BiVO_4$.

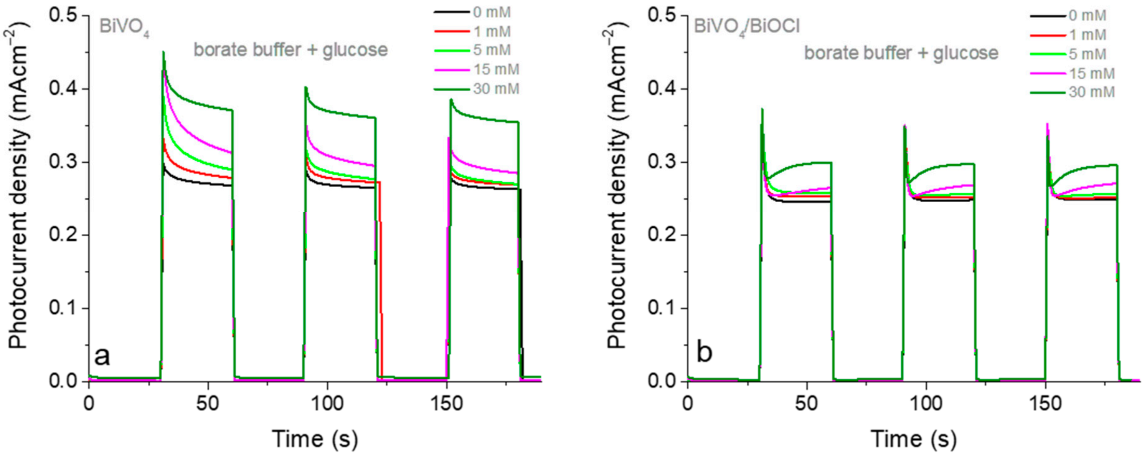

Figure S2. Chronoamperograms of $BiVO_4$ (a) and $BiVO_4$/BiOCl (b) coatings recorded in 0.2 M borate buffer without and containing 1, 5, 15 and 30 mM of glucose in the dark and under light illumination with 30s intervals. Applied potential 1.2 V vs Ag/AgCl.

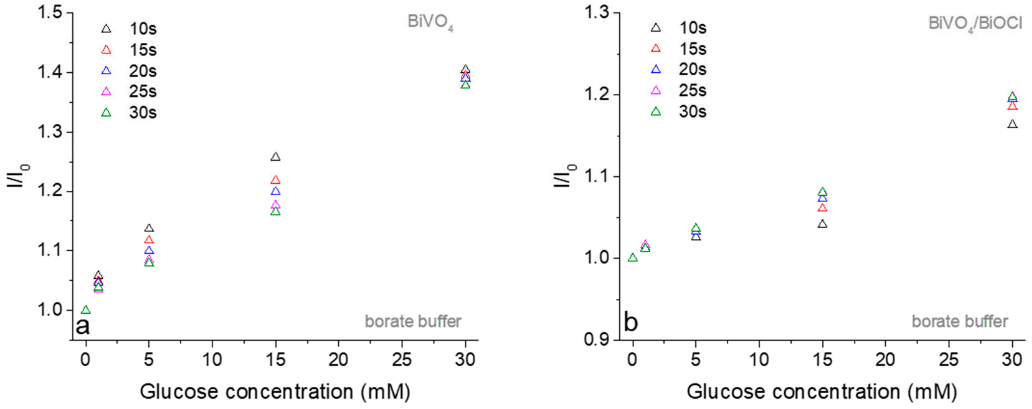

Figure S3. I/I_0 vs glucose concentration of $BiVO_4$ (a) and $BiVO_4$/BiOCl (b) samples.

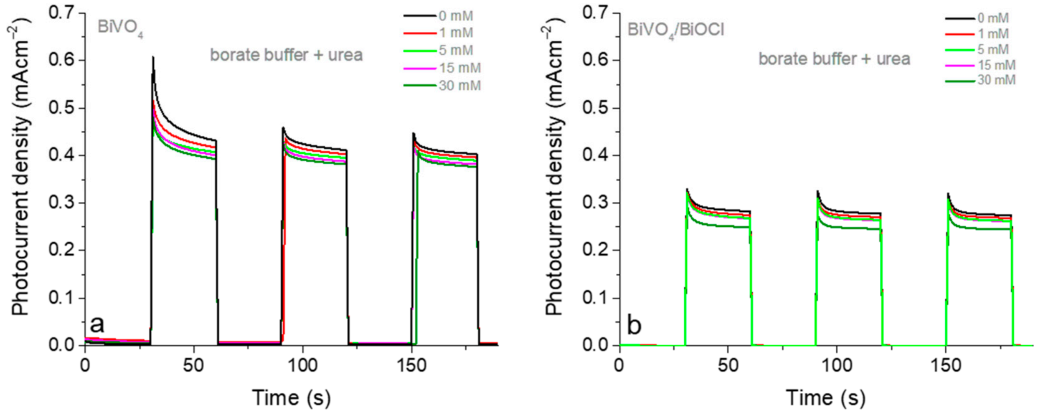

Figure S4. Chronoamperograms of $BiVO_4$ (a) and $BiVO_4$/BiOCl (b) coatings recorded in 0.2 M borate buffer without and containing 1, 5, 15 and 30 mM of urea in the dark and under light illumination with 30s intervals. Applied potential 1.2 V vs Ag/AgCl.

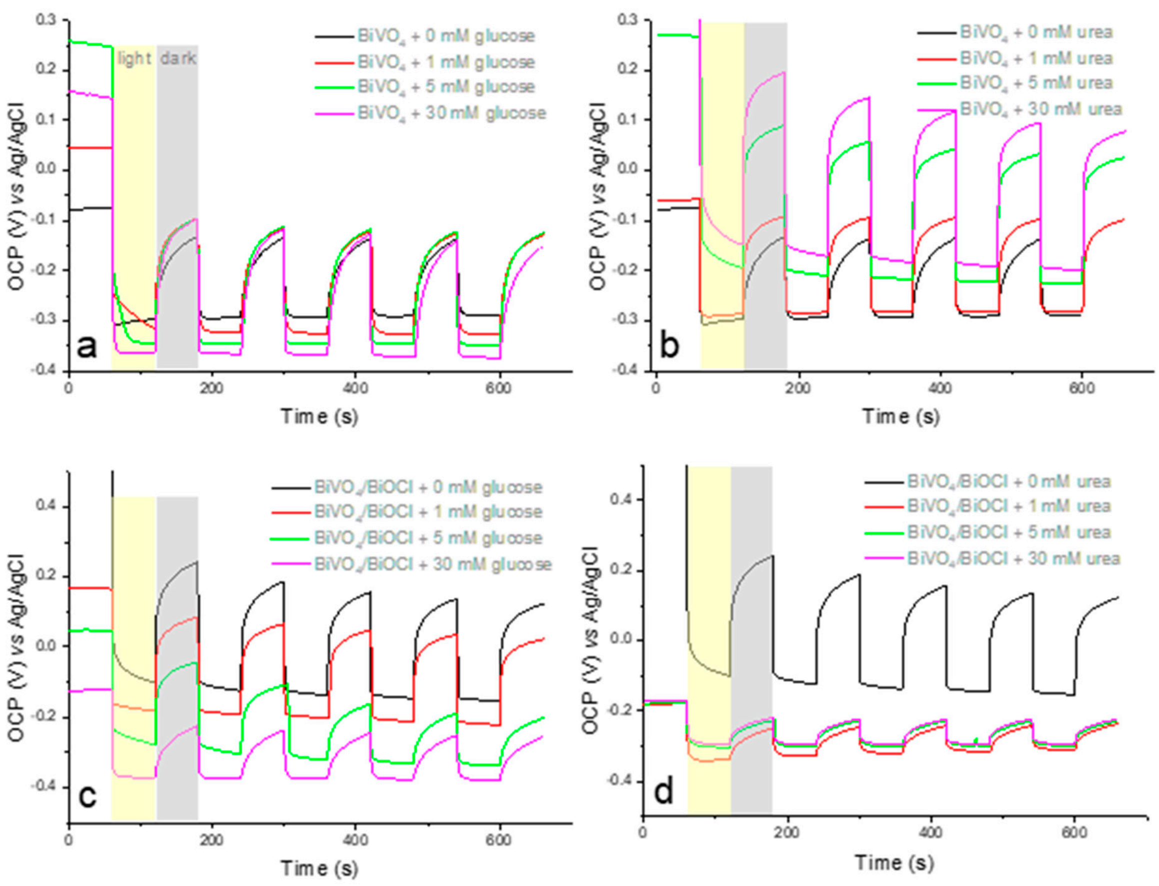

Figure S5. Variation of the open circuit potential (OCP) of $BiVO_4$ (a, b) and $BiVO_4$/BiOCl (c, d) coatings in 0.2 M borate buffer without and with 1, 5 and 30 mM of glucose (a, c) or urea (b, d) under illumination

Table S1. Linear regression equations, correlation coefficients and LOD of $BiVO_4$ and $BiVO_4/BiOCl$ samples.

Time	$BiVO_4$		
	Equation	R^2	LOD, μM
10	$I/I_0 = 0.0127\ C + 1.042$	0.95	1.692
15	$I/I_0 = 0.1237\ C + 1.030$	0.98	0.173
20	$I/I_0 = 0.0122\ C + 1.022$	0.99	1.752
25	$I/I_0 = 0.0120\ C + 1.013$	0.99	1.788
30	$I/I_0 = 0.0119\ C + 1.011$	0.98	1.804
Time	$BiVO_4/BiOCl$		
	Equation	R^2	LOD, μM
10	$I/I_0 = 0.0050\ C + 0.993$	0.9	3.103
15	$I/I_0 = 0.0058\ C + 0.999$	0.94	2.701
20	$I/I_0 = 0.0062\ C + 1.000$	0.97	2.534
25	$I/I_0 = 0.0063\ C + 1.002$	0.99	2.486
30	$I/I_0 = 0.0063\ C + 1.001$	0.99	2.474
